# Multimodal CT and MRI Radiomics Integrated with Clinical Models Predict Pathological Complete Response in ESCC Following Neoadjuvant Immunochemotherapy

**DOI:** 10.3390/tomography11110130

**Published:** 2025-11-19

**Authors:** Longgao Liu, Chufeng Zeng, Lizhi Liu, Shumin Zhou, Weihua Wu, Peng Lin, Jianhua Fu, Tiehua Rong, Xu Zhang, Xiaodong Su

**Affiliations:** 1 Sun Yat-Sen University Cancer Center, 651 Dongfeng Road East, Guangzhou 510060, China; liulg@sysucc.org.cn (L.L.);; 2 Collaborative Innovation Center for Cancer Medicine, State Key Laboratory of Oncology in South China, Sun Yat-Sen University Cancer Center, Guangzhou 510060, China; 3 Guangdong Provincial Clinical Research Center for Cancer, Sun Yat-Sen University Cancer Center, Guangzhou 510060, China

**Keywords:** radiomics, neoadjuvant immunochemotherapy, esophageal squamous cell carcinoma, pathological complete response, multimodal

## Abstract

Background: This research focused on evaluating the utility of multimodal radiomics integrated with machine learning to predict pathological complete response (pCR) in a prospective cohort of esophageal squamous cell carcinoma (ESCC) patients undergoing neoadjuvant immunochemotherapy (nICT). Methods: We retrospectively analyzed prospectively collected trial data from 66 ESCC patients. Radiomic features were extracted from computed tomography (CT) and magnetic resonance imaging (MRI) images. Four machine learning algorithms—Random Forest (RF), logistic regression, Support Vector Machine, and Extreme Gradient Boosting (XGBoost)—were applied with leave-one-out cross-validation to predict pCR after nICT. The predictive performance of the models was evaluated using receiver operating characteristic curve analysis. Results: In total, 851 features were identified. Among the four machine learning algorithms, the XGBoost machine learning method demonstrated the best model performance across CT, MRI, and clinical feature-based models. Furthermore, the integrated model demonstrated superior performance compared to individual models based solely on CT, MRI, or clinical features across all machine learning algorithms. Among these, the XGboost-based integrated model achieved the highest performance on the test set, with an AUC of 0.961, a TPR of 84.2%, a TNR of 95.7%, a PPV 88.9% of and a NPV of 93.8%. Decision curve analysis validated the model’s robust clinical utility, with calibration curves demonstrating strong concordance between predicted and observed therapeutic responses. Conclusions: The study demonstrates the potential for predicting pCR in patients with ESCC treated with standardized neoadjuvant chemotherapy and PD-1 inhibitors using machine learning methods that integrate multimodal CT and MRI images with clinical features.

## Simple Summary

This research developed a noninvasive predictive model for pathologic complete response (pCR) in esophageal squamous cell carcinoma (ESCC) patients undergoing neoadjuvant immunochemotherapy (nICT). By integrating CT and MRI radiomics with clinical data from prospective cohorts, the model demonstrated the potential for predicting pCR. Its promising performance supports its use as a clinical decision-support tool for personalized treatment and reducing the need for invasive procedures.

## 1. Introduction

Esophageal cancer is a major global health challenge and ranks as the seventh leading cause of cancer-related deaths worldwide [1]. In China, esophageal squamous cell carcinoma (ESCC) is the predominant histological subtype, accounting for most esophageal cancer cases [2]. Surgical resection is the primary treatment for locally advanced ESCC; however, patients undergoing surgery alone face high rates of recurrence and metastasis, resulting in poor survival outcomes [3,4]. To reduce recurrence and improve survival, neoadjuvant chemoradiotherapy (nCRT) combined with surgery is recommended as the standard treatment for locally advanced ESCC [4]. Nevertheless, owing to the toxicity of neoadjuvant radiotherapy and the increased risk of perioperative complications, there is a need for alternative and more effective treatment options [5].

With advancements in immunotherapy for esophageal cancer, numerous immunotherapy-related clinical studies are underway [6,7,8,9]. Neoadjuvant immunochemotherapy (nICT) has emerged as a promising treatment approach [10,11,12,13]. Studies have shown that compared with neoadjuvant chemotherapy (nACT) alone, nICT significantly improves the pathological complete response (pCR) rate and enhances patient survival [14]. Additionally, nICT offers comparable disease-free survival and overall survival outcomes to nCRT but with fewer side effects [15]. Consequently, the role of nICT in treating esophageal cancer is likely to expand. Predicting the response of ESCC patients to nICT is essential for guiding treatment decisions, as achieving pathological complete response (pCR) after neoadjuvant therapy is strongly associated with improved survival outcomes [16,17]. However, since pCR is determined histologically from postoperative specimens, a noninvasive preoperative method for predicting pCR is needed to guide personalized treatment strategies.

Radiomics, which involves high-throughput extraction of quantitative features from medical images, shows promise as a noninvasive tool for predicting pCR. Recent studies have highlighted the utility of radiomics in predicting tumor diagnosis, therapeutic response, and survival across various cancers [18,19], offering potential guidance for personalized treatment in ESCC. For instance, Li et al. developed machine learning models based on delta CT imaging features to predict pathological response to therapy in patients with ESCC following nICT, achieving an area under the receiver operating characteristic (ROC) curve (AUC) of 0.848 [20]. Liu et al. developed and validated a machine learning model based on MR radiomics to accurately predict pCR after nCRT in patients with ESCC [21]. These studies have revealed the significant potential of CT and MRI in predicting the efficacy of neoadjuvant therapy for esophageal cancer. However, research on multimodal radiomics features for predicting pathological response after nICT in patients with ESCC remians limited.

Therefore, this study focused on evaluating the utility of multimodal radiomics integrated with machine learning to predict pCR in a prospective cohort of patients with ESCC treated with nICT. Furthermore, the study evaluated and compared the predictive capabilities of models employing four distinct machine learning algorithms.

## 2. Materials and Methods

### 2.1. Patients

The studies involving humans were approved by the Ethics Committee of Sun Yat-sen University Cancer Center (approval number: SL-B2021-335-03) on 7 March 2023.

**Figure 1:**
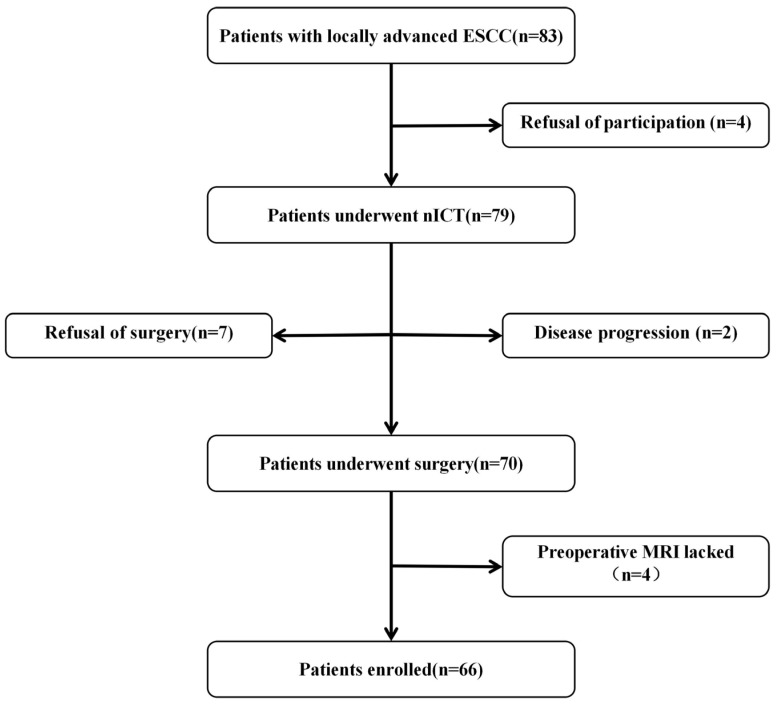
The flowchart of patients’ enrollment. nICT neoadjuvant immunochemotherapy, ESCC esophageal squamous cell carcinoma.

The patient selection flowchart is presented in Figure 1. This study reanalyzed data from prospective trials (ChiCTR2200061094, ChiCTR2000029807) that enrolled patients with ESCC who received standard neoadjuvant immunotherapy combined with chemotherapy, followed by esophagectomy, at Sun Yat-sen University Cancer Center from January 2020 to January 2024. Inclusion criteria were (1) stage II or III locally advanced resectable ESCC diagnosed before enrollment (2) no distant organ metastases or cervical lymph node metastases prior to enrollment (3) no secondary primary tumors (4) an Eastern Cooperative Oncology Group (ECOG) performance status score 0 or 1 (5) no prior exposure to anticancer therapy, including chemotherapy, radiotherapy, targeted therapy, and immunotherapy. The exclusion criteria included (1) poor image quality due to significant artifacts (2) incomplete clinical or pathological data.

Each participant underwent three cycles of chemotherapy and PD-1 blockade. During each cycle, patients received a flat intravenous dose of camrelizumab (200 mg) and nab-paclitaxel (260 mg/m^2^) on day 1. Additionally, capecitabine was administered orally at 1250 mg/m^2^ twice daily from day 1 to 14, with the cycle repeated every 3 weeks. After the final chemotherapy session, patients underwent enhanced computed tomography (CT) and magnetic resonance imaging (MRI) scans. MRI scans were recommended but not mandatory; hence, patients without preoperative MRI scans were excluded from the study. Ultimately, 66 patients with ESCC with complete preoperative enhanced CT and MRI scans were included.

McKeown esophagectomy was performed within 4–6 weeks after nICT. PCR was defined as the absence of residual cancer in the primary tumor or lymph nodes in the postoperative pathology specimen. Clinical and demographic data—including sex, age, height, weight, BMI, family history, smoking and alcohol use history, comorbidities, tumor location, pretreatment blood markers (absolute values of albumin, hemoglobin, platelets, red blood cells, white blood cells, neutrophils, lymphocytes, monocytes, and C-reactive protein), clinical T and N stages, and the interval between the last immunochemotherapy session and surgery—were manually extracted.

### 2.2. Radiomic Feature Extraction and Model Construction

**Figure 2:**
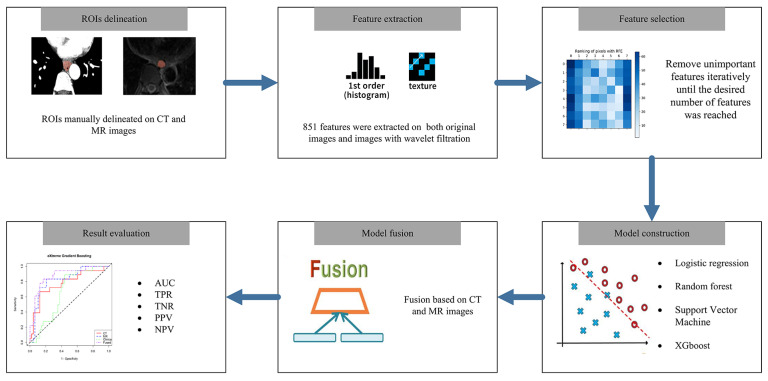
Treatment response prediction workflow.

A schematic representation of the treatment response prediction workflow used in this study is provided in Figure 2. The region of interest (ROI) was manually selected as the slice with the maximum axial diameter of the tumor and was delineated on ITK-SNAP (version 3.8.0) by a senior thoracic surgeon, with verification by a senior radiologist. The ROI was delineated and analyzed in the arterial phase of CT images and the T2-weighted MRI images of each selected patient. Original and wavelet radiomic features were then extracted from CT and MRI images using Pyradiomics (version 3.1.0) in Python 3.6.

Five CT scanners, including Discovery CT750 HD (GE Healthcare, Waukesha, WI, USA), AQUILION TSX-101A (Canon Medical Systems, Otawara, Tochigi, Japan), SOMATOM Force (Siemens Healthineers, Erlangen, Germany), Brilliance iCT (Philips Healthcare, Best, Netherlands), and uCT 78 (United Imaging Healthcare, Shanghai, China), were used for imaging. Scans covered the region from the thoracic inlet to 2–7 cm below the lower costal margin, adjusted according to lesion extent. The tube voltage was set at 120 kV, with tube current modulation ranging from 100 to 300 mA. The field of view was between 400 mm and 500 mm, with a matrix size of 512 × 512 pixels. Slice thickness and interval were set at 5 mm; in contrast, thin-slice reconstruction parameters were set to a thickness of 1.25 mm. A high-pressure injector delivered a nonionic iodinated contrast agent at 3.0 mL/s, with images acquired in the arterial and venous phases at delays of 25 s and 55 s, respectively. Detailed multi-sequence MRI acquisition protocols and MRI parameters are available in Table S1.

To account for variations in in-plane resolution and acquisition protocols, the enrolled images including CT and MR were resampled to a uniform voxel spacing. The images were resampled to 1 × 1 mm^2^ in-plane resolution to ensure consistency across scans while preserving in-plane structural details. Z-score normalization was implemented before extracting traditional features. A total of 851 radiomic features were extracted from both CT and MRI images. Four models were subsequently developed: CT, MRI, clinical, and fusion models, with the fusion model combining features from all three models. The model-building process using machine learning algorithms included several steps. First, randomly removing one feature from each pair of strongly correlated features (correlation coefficient > 0.9). Following this, the support vector machines with recursive feature elimination (SVM-RFE) were used, and cross-validation was applied for recursive feature elimination, beginning with all remaining features and iteratively removing less significant ones until the target number of features was reached. To avoid overfitting due to excessive feature selection, in accordance with the one-tenth rule, we constrained the number of radiomic features to 4–7 and subsequently constructed distinct models for each feature subset.

Following feature selection, the models were trained using four machine learning algorithms: logistic regression, Support Vector Machine, Random Forest (RF), and extreme Gradient Boosting (XGBoost). The leave-one-out cross-validation method was implemented, in which each patient was used as the test set once; in contrast, the remaining 65 patients served as the training set. This process was repeated for all 66 patients, and the average prediction result was used as the final prediction for the test set. Using each algorithm, four models were built for CT, MRI, and clinical features. The model with the highest AUC in the test set was selected as the final model.

### 2.3. Model Fusion

All models discussed were developed and trained using features derived from a single modality—CT, MRI, or clinical data—using different machine learning algorithms. A fusion approach was also implemented, where the output probabilities and selected thresholds from the CT, MRI, and clinical models were combined. In this process, the probabilities were weighted and aggregated to produce the final output, and classification was performed based on these weighted thresholds to derive the fusion outcome. Notably, the fusion approach was restricted to models using the same machine learning algorithm, as models with different algorithms were not merged. The predictive performances of single-modality models (CT-only, MRI-only, and clinical-only) were evaluated and compared with the fusion model incorporating CT, MRI, and clinical features.

### 2.4. Statistical Analysis

Radiomic features were extracted using Pyradiomics (version 3.1.0) in Python 3.6; in contrast, feature selection, model training, validation, and plotting were conducted in R 4.4.1. Statistical analyses, including *t*-tests, Pearson’s correlation analysis, chi-squared tests, and Fisher’s exact tests, were also performed in R 4.4.1. The ROC curve was plotted to assess model performance, with metrics such as AUC, true positive rate (TPR), true negative rate (TNR), positive predictive value (PPV), and negative predictive value (NPV) calculated for further evaluation. Continuous variables are presented as mean ± standard deviation and categorical variables as counts (%). Relationships between variables were examined using Pearson’s correlation analysis. Comparisons of AUC values were conducted using the DeLong test. Statistical significance was set at *p* < 0.05.

## 3. Results

### 3.1. Baseline Characteristics

A total of 66 patients were included in the study. Among them, 17 patients (25.8%) had stage II disease, and 49 patients (74.2%) had stage III disease. Most were male and presented with moderately differentiated tumors. In total, 18 patients achieved pCR. Baseline characteristics are summarized in Table 1.

### 3.2. Performance of Four Machine Learning Radiomic and Clinical Models

We developed CT, MRI, and clinical feature models using four machine learning algorithms. For each algorithm, the best model was constructed for the CT, MRI, and clinical feature sets, as summarized in Table 2. As summarized in Table 2, the XGBoost machine learning method demonstrated the best model performance across CT, MRI, and clinical feature-based models. For CT features, radiomics model demonstrated superior performance, achieving an AUC of 0.903 (95% CI: 0.8226–0.983). In MRI features, radiomics model showed optimal performance with an AUC of 0.846 (95% CI: 0.7502–0.9419). The clinical model exhibited moderate performance, with an AUC of 0.668 (0.5261–0.8096). By using different machine learning methods, the AUC value ranges from 0.744 to 0.903 for CT models, from 0.669 to 0.846 for MRI models, and from 0.613 to 0.668 for clinical models. The CT and MRI models demonstrated superior performance to the clinical models, with a significant difference (*p* < 0.01). Notably, there was no statistically significant difference in AUC values between the CT and MRI models. Specifically, the CT model with the highest AUC (0.903) included four features. The MRI model with the highest AUC (0.846) included five features. And the clinical model with the highest AUC (0.668) incorporated seven features. The features of the three best models are detailed in Table 3. Additionally, four models for the CT, MRI, and clinical feature sets, differentiated by the number of features, were trained and summarized in Table S2.

### 3.3. Fused Models

**Figure 3:**
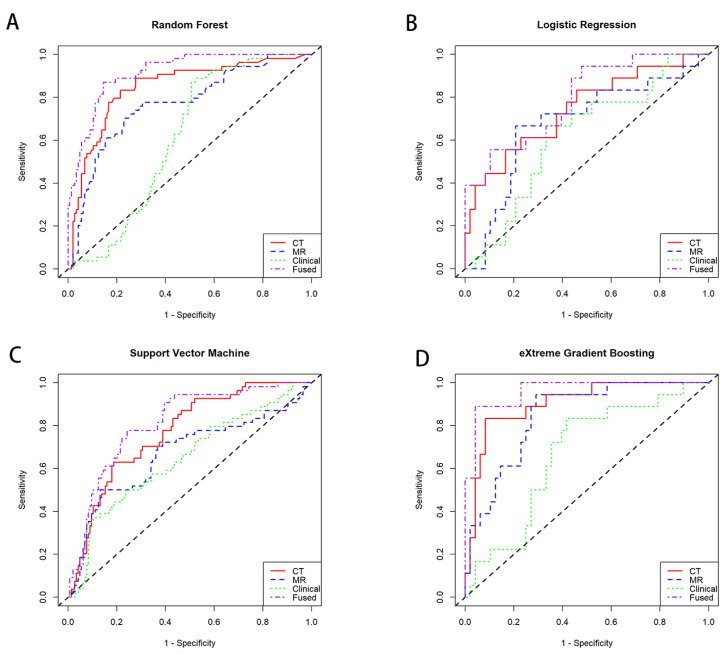
The receiver operating characteristic (ROC) curve for the models. CT model, MRI model, clinical model and CT + MRI + clinical fused model are represented by the red, blue, green and purple lines, respectively. (**A**) Random Forest, (**B**) Logistic Regression, (**C**) Support Vector Machine, (**D**) eXtreme Gradient Boosting.

Using four machine learning methods, we developed a prediction model that combined CT, MRI, and clinical features. For each method, the best CT, MRI, and clinical feature models were fused to create the prediction model. As shown in Table 2, the integrated model demonstrated superior performance compared to individual models based solely on CT, MRI, or clinical features across all machine learning algorithms. Among these, the XGboost-based integrated model achieved the highest performance on the test set, with an AUC of 0.961, a TPR of 84.2%, a TNR of 95.7%, a PPV 88.9% of and a NPV of 93.8% (Table 2). This exceeded the AUC of models based solely on CT, MRI, or clinical features (Figure 3). The DeLong test comparing the performance between them showed a significant difference (*p* < 0.001) (Figure S1). Subsequently, we conducted decision curve analysis (DCA) to evaluate the influence of the optimal CT, MRI, clinical, and fusion models on clinical treatment decision-making. As illustrated in Figure S2, the XGBoost-based fusion model provided greater net benefit compared to the treat-all or treat-none strategies within a threshold probability range of 0% to 100% in the test cohort. Furthermore, the calibration curves for the XGBoost-based fusion model demonstrated strong concordance between predicted and actual treatment responses (Figure S3). These findings suggest that fusing features from multiple models can considerably improve prediction accuracy. The section may be divided by subheadings. It should provide a concise and precise description of the experimental results, their interpretation, as well as the experimental conclusions that can be drawn.

## 4. Discussion

Our study demonstrated promising results in predicting pCR in patients with ESCC treated with nICT by applying multiple machine learning methods that integrate multimodal CT and MRI features with clinical data. To our knowledge, this is the first study to evaluate the utility of combining multimodal radiomics from CT and MRI with machine learning to predict pCR in a prospective cohort of patients with ESCC undergoing nICT.

Recent studies have developed predictive models for pCR using clinical characteristics, imaging features, and biological markers [22,23,24,25,26,27]. For instance, Wang et al. created machine learning models to predict primary pCR and total pCR of ESCC patients who underwent nCRT based on CT radiomics, achieving AUCs of 0.891 and 0.814 on the test set [22]. Hu et al. employed deep learning with CT images to build an optimal model based on features from ResNet50, achieving an AUC of 0.805 (95% confidence interval, 0.696–0.913) and an accuracy of 77.1% (65.6–86.3%) on the testing cohort [23]. Additionally, Lu et al. established MRI-based radiomics models for predicting pathological response after nACT in patients with ESCC, achieving AUCs of 0.851 on the training set and 0.831 on the validation set [26]. However, these studies were largely retrospective and often limited to single-modality imaging or clinical data. In contrast, our study used a prospective approach, which enhances reliability and clinical applicability. By using real-time data collected before treatment, we reduced biases often associated with retrospective analyses.

In our study, we evaluated four machine learning models and found that the XGBoost model achieved the highest performance for both the single-modality CT model (AUC of 0.903) and the fusion model integrating CT, MRI, and clinical data (AUC of 0.961). The CT + MRI + clinical fusion model consistently outperformed the CT-only and MRI-only models across all machine learning methods, demonstrating the potential of feature fusion to improve prediction accuracy. Previous multimodal radiomics studies, such as that by Liu et al., have demonstrated improved predictive performance by integrating CT, T2-weighted MRI, and diffusion-weighted imaging (DWI) of patients treated with neoadjuvant chemoradiotherapy, achieving an AUC of 0.868 [27]. In contrast, our multimodal model incorporated CT, MRI, and clinical features in a cohort of patients treated with neoadjuvant chemotherapy combined with immunotherapy. This specific treatment context represents a novel and clinically relevant scenario, as immune-related responses may alter tumor biology and imaging phenotypes compared to conventional chemoradiotherapy. Our findings therefore extend the application of multimodal radiomics to the immunochemotherapy setting, highlighting its potential for individualized response prediction. Despite these promising results, further validation is needed. Large-scale, multicenter clinical studies are essential to confirm the robustness and generalizability of our predictive model across diverse patient populations.

Several studies have improved prediction results by combining imaging and clinical features during model training [28,29,30]. Although no significant differences were observed between the clinical characteristics of patients with pCR and non-pCR, we still incorporated patient clinical characteristics into our model. These factors may still influence patient outcomes by affecting treatment tolerance and immune status; hence, including them in the study is advisable.

This study had notable strengths. First, we used multimodal radiomics to integrate CT and MRI features, capturing a broader range of tumor characteristics that enhance predictive accuracy. To our knowledge, few studies have used radiomics to predict therapeutic outcomes of neoadjuvant chemotherapy combined with immunotherapy for esophageal cancer, especially using multimodal imaging. This gap highlights the novelty and potential clinical implications of our study. Additionally, our cohort consisted of prospectively identified patients who underwent standardized nACT and PD-1 inhibitor treatment, which improves the model’s generalizability.

However, this study has limitations. First, it was a single-center study with a small sample size and no external validation cohort, highlighting the need for multicenter studies to further validate our model. And both CT and MRI are not routinely performed for all ESCC patients, which may affect applicability. Additionally, although pCR is the primary goal of neoadjuvant therapy for esophageal cancer and is linked to long-term survival, it remains unclear whether our model can predict long-term patient outcomes. Finally, we focused only on primary tumors when predicting pCR, not accounting for lymph node involvement, which may have affected model accuracy. Further studies should consider including metastatic lymph nodes in the prediction model.

## 5. Conclusions

Our study achieved promising results in predicting pCR in patients with ESCC treated with standardized nICT by applying multimodality methods that integrate multimodal CT and MRI features along with clinical data. Notably, the model developed using the XGBoost machine learning approach demonstrated superior potential in prediction when compared with Random Forest, Logistic Regression, and Support Vector Machine algorithms. This approach provides a noninvasive and efficient method for pCR prediction following nICT and supports precise, individualized treatment strategies.

## Figures and Tables

**Table 1:** Baseline characteristics of selected patients.

Characteristics	Overall	Non-PCR	PCR	*p* Value
N	66	48	18	
Sex (%)				1.000
Male	52 (78.8)	38 (79.2)	14 (77.8)	
Female	14 (21.2)	10 (20.8)	4 (22.2)	
Age	59.20 ± 6.57	59.96 ± 6.32	57.17 ± 6.97	0.125
Family history (%)				0.697
No	51 (77.3)	36 (75.0)	15 (83.3)	
Yes	15 (22.7)	12 (25.0)	3 (16.7)	
BMI	22.08 (3.22)	22.16 (3.51)	21.87 (2.34)	0.748
Alcohol drinking (%)				0.297
Never	38 (57.6)	30 (62.5)	8 (44.4)	
Former or Current	28 (42.4)	18 (37.5)	10 (55.6)	
Smoking (%)				0.939
Never	28 (42.4)	21 (43.8)	7 (38.9)	
Former or Current	38 (57.6)	27 (56.2)	11 (61.1)	
Comorbidity (%)				0.466
No	45 (68.2)	31 (64.6)	14 (77.8)	
Yes	21 (31.8)	17 (35.4)	4 (22.2)	
Difference (%)				0.084
Poorly differentiated	20 (30.3)	11 (22.9)	9 (50.0)	
Moderately differentiated	44 (66.7)	35 (72.9)	9 (50.0)	
Well-differentiated	2 (3.0)	2 (4.2)	0 (0.0)	
Location (%)				0.299
Lower thoracic	33 (50.0)	26 (54.2)	7 (38.9)	
Middle thoracic	31 (47.0)	20 (41.7)	11 (61.1)	
Upper thoracic	2 (3.0)	2 (4.2)	0 (0.0)	
Clinical T stage (%)				0.800
T2	18 (27.3)	14 (29.2)	4 (22.2)	
T3	48 (72.7)	34 (70.8)	14 (77.8)	
Clinical N stage (%)				0.257
N0	4 (6.1)	4 (8.3)	0 (0.0)	
N1	27 (40.9)	21 (43.8)	6 (33.3)	
N2	35 (53.0)	23 (47.9)	12 (66.7)	
Clinical stage (%)				0.473
II	17 (25.8)	14 (29.2)	3 (16.7)	
III	49 (74.2)	34 (70.8)	15 (83.3)	
ALB	44.48 ± 3.27	44.37 ± 3.63	44.76 ± 2.07	0.671
WBC	7.41 ± 1.71	7.36 ± 1.51	7.52 ± 2.20	0.741
NEU	4.70 ± 1.36	4.60 ± 1.30	4.98 ± 1.51	0.310
LY	2.03 ± 0.60	2.08 ± 0.53	1.88 ± 0.74	0.207
MO	0.47 ± 0.16	0.45 ± 0.12	0.50 ± 0.24	0.247
HGB	142.50 ± 16.06	141.83 ± 16.62	144.28 ± 14.76	0.586
PLT	271.98 ± 83.06	277.27 ± 74.56	257.89 ± 103.54	0.403
RBC	4.74 ± 0.55	4.76 ± 0.60	4.69 ± 0.38	0.673
CRP	7.20 ± 9.92	7.12 ± 10.37	7.41 ± 8.90	0.917
Time from completion of nICT to surgery	37.82 ± 11.60	38.29 ± 12.17	36.56 ± 10.14	0.592

BMI body mass index, ALB albumin, WBC white blood cell, NEU neutrophil count, LY lymphocyte count, MO monocyte count, HGB hemoglobin, PLT platelet count, RBC red blood cell, CRP C-reactive protein, nICT neoadjuvant immunochemotherapy.

**Table 2:** The prediction performance of different machine learning models was assessed.

RF	AUC	TPR	TNR	PPV	NPV
CT	0.846	0.690	0.840	0.537	0.910
MRI	0.768	0.628	0.826	0.500	0.889
Clinical	0.613	0.388	0.909	0.870	0.486
Fused	0.916	0.691	0.946	0.870	0.854
LR	AUC	TPR	TNR	PPV	NPV
CT	0.744	0.556	0.833	0.556	0.833
MRI	0.677	0.522	0.860	0.667	0.771
Clinical	0.620	0.429	0.842	0.667	0.667
Fused	0.792	0.643	0.827	0.500	0.896
SVM	AUC	TPR	TNR	PPV	NPV
CT	0.769	0.595	0.801	0.407	0.896
MRI	0.669	0.613	0.790	0.352	0.917
Clinical	0.646	0.476	0.751	0.185	0.924
Fused	0.810	0.612	0.839	0.556	0.868
XGBoost	AUC	TPR	TNR	PPV	NPV
CT	0.903	0.789	0.936	0.833	0.917
MRI	0.846	0.625	0.840	0.556	0.875
Clinical	0.668	0.409	0.795	0.500	0.729
Fused	0.961	0.842	0.957	0.889	0.938

SVM Support Vector Machine, LR Logistic Regression, RF Random Forest, XGBoost eXtreme Gradient Boosting, TPR true positive rate, TNR true negative rate, PPV positive predictive value, NPV negative predictive value.

**Table 3:** Finalized radiomics features and clinical features identified and incorporated in the best model construction.

CT feature names
wavelet.HLH_glrlm_LongRunLowGrayLevelEmphasis wavelet.LLH_firstorder_Maximum wavelet.HHH_firstorder_Median wavelet.LLL_glcm_MCC wavelet.LLL_glcm_Correlation
MRI feature names
wavelet.LHL_firstorder_Skewness wavelet.LLL_firstorder_Median wavelet.LHH_firstorder_Median wavelet.LLL_firstorder_Minimum
Clinical feature names
LY
difference
comorbidity
WBC
cN
clinical stage ALB

LY lymphocyte count, WBC white blood cell, cN clinical N stage, ALB albumin.

## Data Availability

The datasets used and analyzed during the current study are available from the corresponding author on reasonable request.

## Supplementary Materials

The following supporting information can be downloaded at: https://www.mdpi.com/article/10.3390/tomography11110130/s1, Figure S1: Comparison of different models’ performances in the testing cohort by DeLong test; Figure S2: Decision curve analysis of models in the testing cohort; Figure S3: Calibration curves of models in the testing cohort; Table S1: Magnetic resonance imaging scanning parameters for the patients; Table S2: The prediction performance of different machine learning (ML) models was evaluated.
