# Supplementary material for: Multimodal CT and MRI Radiomics Integrated with Clinical Models Predict Pathological Complete Response in ESCC Following Neoadjuvant Immunochemotherapy
Source: Tomography. 2025 Nov 19;11(11):130. doi: 10.3390/tomography11110130 (PMC12656131; doi:10.3390/tomography11110130)
Supplement: Supplementary file 1 [file tomography-11-00130-s001.zip › tomography-3919540-Supplementary.pdf]

Multimodal CT and MRI Radiomics Integrated with Clinical Models Predict Pathological Complete Response in ESCC Following Neoadjuvant Immunochemotherapy

SUPPLEMENTARY MATERIA

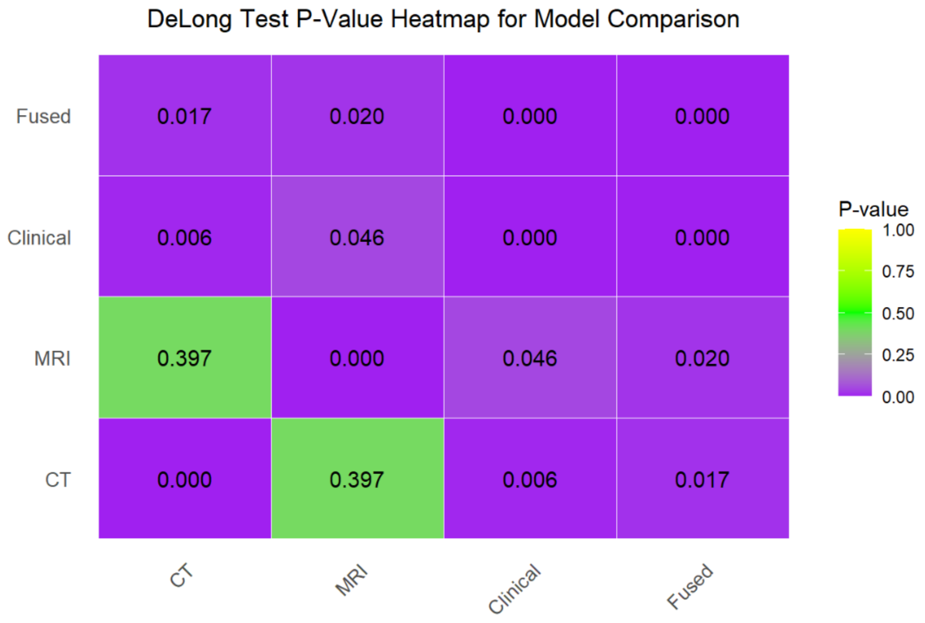

Figure S1 Comparison of different models' performances in the testing cohort by DeLong test

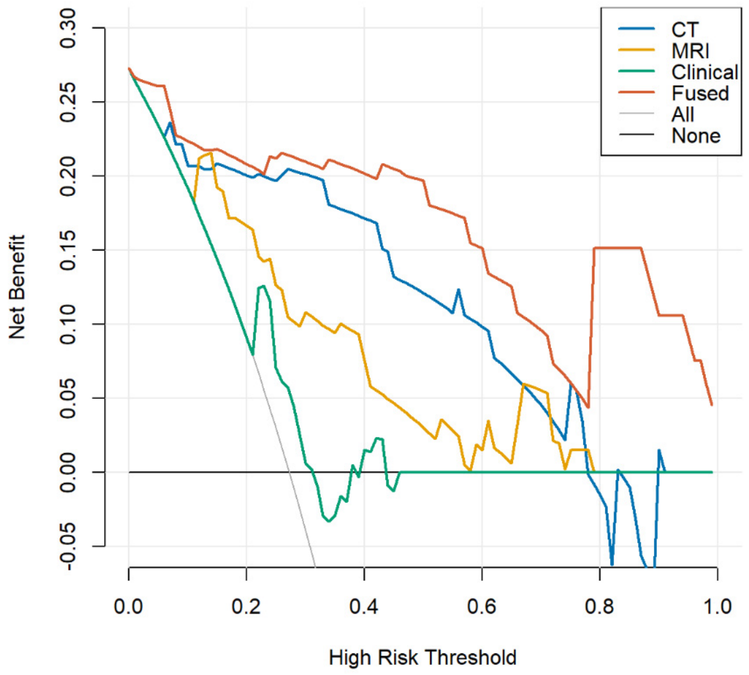

Figure S2 Decision curve analysis of models in the testing cohort.

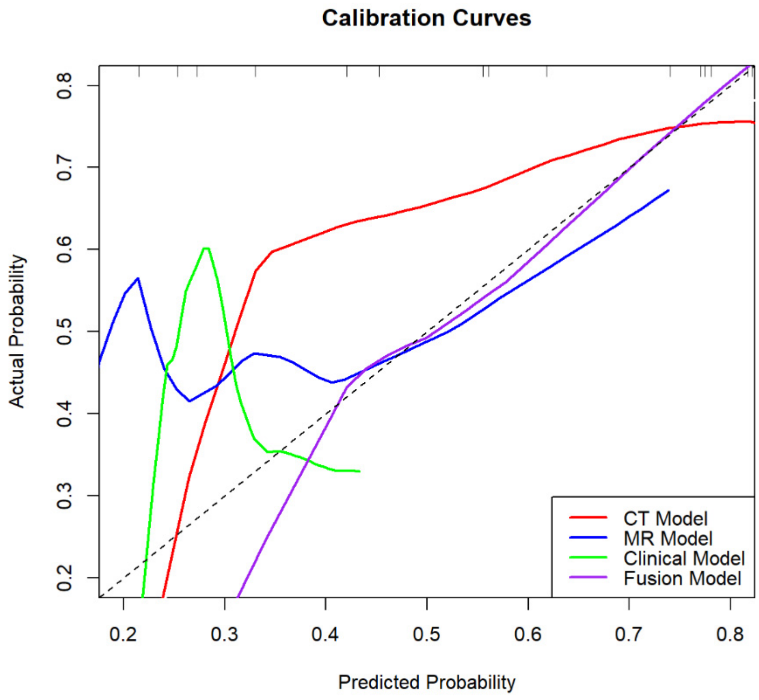

Figure S3 Calibration curves of models in the testing cohort.

TableS1 Magnetic resonance imaging scanning parameters for the patients

**Scanner**	**Sequence**	**TR/TE (ms)**	**FOV (mm)**	**Matrix**	**Slice Thickness (mm)**	**Slice Gap (mm)**	**Slices**	**Flip Angle**	**Acquisition Time (min)**	**Scans**
United Imaging 3·0T (China)	T2WI	3600/74·34	340×340	336×335	5	1	24	90°	3min05s	
	DWI-ADC	3597/67·2	350×350	350×190	6	1	24	90°	1min33s	
	T1+C	4·3/1·99	340×340	336×335	0·67	0	204	10°	9min58s	8
GE 3·0T (USA)	T2WI	3912/107·64	380×380	416×256	5	1	28	111°	1min59s	
	DWI-ADC	4168/60·2	380×380	128×160	5	1	48	-	1min40s	
	T1+C	4·3/1·7	360×360	256×320	1	0	204	5°	9min50s	8

Abbreviations: FOV, field of view; TR, repetition time; TE, echo time; T1+C, contrast-enhanced T1-weighted imaging; T2WI, T2-weighted imaging.

TableS2 The prediction performance of different machine learning (ML) models was evaluated.

ML Type	Model type	Feature Number	AUC	TPR	TNR	PPV	NPV
RF	MR Radiomics	7	0.766	0.683	0.834	0.519	0.910
RF	MR Radiomics	6	0.759	0.629	0.804	0.407	0.910
RF	MR Radiomics	5	0.723	0.508	0.842	0.611	0.778
RF	MR Radiomics	4	0.768	0.628	0.826	0.500	0.889
RF	CT Radiomics	7	0.830	0.833	0.827	0.463	0.965
RF	CT Radiomics	6	0.827	0.721	0.852	0.574	0.917
RF	CT Radiomics	5	0.846	0.690	0.840	0.537	0.910
RF	CT Radiomics	4	0.823	0.705	0.851	0.574	0.910
RF	Clinical	7	0.574	0.308	0.744	0.370	0.688
RF	Clinical	6	0.557	0.308	0.750	0.444	0.625
RF	Clinical	5	0.536	0.319	0.797	0.704	0.438
RF	Clinical	4	0.613	0.388	0.909	0.870	0.486
RF	Combined		0.916	0.691	0.946	0.870	0.854
LR	MR Radiomics	7	0.638	0.429	0.800	0.500	0.750
LR	MR Radiomics	6	0.620	0.450	0.804	0.500	0.771
LR	MR Radiomics	5	0.654	0.500	0.800	0.444	0.833
LR	MR Radiomics	4	0.677	0.522	0.860	0.667	0.771
LR	CT Radiomics	7	0.744	0.556	0.833	0.556	0.833
LR	CT Radiomics	6	0.653	0.563	0.820	0.500	0.854
LR	CT Radiomics	5	0.708	0.643	0.827	0.500	0.896
LR	CT Radiomics	4	0.735	0.588	0.837	0.556	0.854
LR	Clinical	7	0.620	0.429	0.842	0.667	0.667
LR	Clinical	6	0.494	0.313	0.740	0.278	0.771
LR	Clinical	5	0.500	0.375	0.741	0.167	0.896

LR	Clinical	4	0.479	0.389	0.771	0.389	0.771
LR	Combined		0.792	0.643	0.827	0.500	0.896
SVM	MR Radiomics	7	0.539	0.322	0.748	0.352	0.722
SVM	MR Radiomics	6	0.610	0.426	0.796	0.481	0.757
SVM	MR Radiomics	5	0.543	0.347	0.752	0.315	0.778
SVM	MR Radiomics	4	0.669	0.613	0.790	0.352	0.917
SVM	CT Radiomics	7	0.709	0.571	0.791	0.370	0.896
SVM	CT Radiomics	6	0.687	0.479	0.793	0.426	0.826
SVM	CT Radiomics	5	0.769	0.595	0.801	0.407	0.896
SVM	CT Radiomics	4	0.756	0.565	0.816	0.481	0.861
SVM	Clinical	7	0.621	0.259	0.725	0.130	0.861
SVM	Clinical	6	0.609	0.185	0.713	0.093	0.847
SVM	Clinical	5	0.602	0.162	0.702	0.111	0.785
SVM	Clinical	4	0.646	0.476	0.751	0.185	0.924
SVM	Combined		0.810	0.612	0.839	0.556	0.868
Xgboost	MR Radiomics	7	0.822	0.556	0.833	0.556	0.833
Xgboost	MR Radiomics	6	0.810	0.500	0.826	0.556	0.792
Xgboost	MR Radiomics	5	0.803	0.600	0.824	0.500	0.875
Xgboost	MR Radiomics	4	0.846	0.625	0.840	0.556	0.875
Xgboost	CT Radiomics	7	0.802	0.733	0.863	0.611	0.917
Xgboost	CT Radiomics	6	0.822	0.667	0.911	0.778	0.854
Xgboost	CT Radiomics	5	0.903	0.789	0.936	0.833	0.917
Xgboost	CT Radiomics	4	0.841	0.706	0.878	0.667	0.896
Xgboost	Clinical	7	0.668	0.409	0.795	0.500	0.729
Xgboost	Clinical	6	0.576	0.333	0.762	0.444	0.667
Xgboost	Clinical	5	0.571	0.400	0.805	0.556	0.688
Xgboost	Clinical	4	0.576	0.400	0.783	0.444	0.750
Xgboost	Combined		0.961	0.842	0.957	0.889	0.938

SVM, Support Vector Machine; LR, Logistic Regression; RF, Random Forest; XGBoost, extreme Gradient Boosting; TPR, true positive rate; TNR, true negative rate; PPV, positive predictive value; NPV, negative predictive value.
